# Sustainability of INFORM: A Complex Team-Based Improvement Intervention in Long-Term Care

**DOI:** 10.1093/geroni/igab046.1444

**Published:** 2021-12-17

**Authors:** Matthias Hoben, Liane Ginsburg, Whitney Berta, James Dearing, Peter Norton, Malcolm Doupe, Janice Keefe, Jude Spiers

**Affiliations:** 1 University of Alberta at Edmonton, Edmonton, Alberta, Canada; 2 York University, York University, Alberta, Canada; 3 University of Toronto, University of Toronto, Ontario, Canada; 4 Michigan State University, Michigan State University, Michigan, United States; 5 University of Calgary, University of Calgary, Alberta, Canada; 6 University of Manitoba, Winnipeg, Manitoba, Canada; 7 Mount Saint Vincent University, Halifax, Nova Scotia, Canada; 8 University of Alberta, Edmonton, Alberta, Canada

## Abstract

Improving Nursing Home Care Through Feedback On perfoRMance Data (INFORM) was a complex, theory-based, three-arm, parallel cluster-randomized trial. In 2015–2016, we successfully implemented two theory-based feedback strategies (compared to a standard approach to feedback) to increase nursing home (NH) care aides’ involvement in formal communications about resident care (formal interactions [FI], the primary outcome). Here, we report the extent to which FI was sustained 2.5 years following withdrawal of intervention supports. We also report on several determinants of sustainability. We analyzed data from 18 NHs (46 units, 529 care aides) in the control group, 19 NHs (60 units, 731 care aides) in the basic assisted feedback group (BAF), and 14 homes (41 units, 537 care aides) in the enhanced assisted feedback group (EAF). We assessed sustainability of FI, using repeated measures, hierarchical mixed models, adjusted for care aide, care unit and facility variables. In EAF, FI scores increased from T1 (baseline) to T2 (end of intervention) (1.30–1.42, p=0.010), remaining stable at T3 (long-term follow-up) (1.39 p=0.065). FI scores in BAF increased from T1 to T2 (1.33–1.44, p=0.003) and continued to increase at T3 (1.49, p<0.001). In the control group, FI did not change from T1 to T2 (1.25–1.24, p=0.909), but increased at T3 (1.38, p=0.003). Better culture, evaluation and fidelity enactment significantly increased FI at long-term follow-up. Theory-informed feedback provides long lasting benefits in care aides' involvement in FI. Greater intervention intensity neither implies greater effectiveness nor sustainability. Modifiable context elements and fidelity enactment may facilitate sustained improvement.

